# A long non-coding RNA, HOTAIR, promotes cartilage degradation in osteoarthritis by inhibiting WIF-1 expression and activating Wnt pathway

**DOI:** 10.1186/s12860-020-00299-6

**Published:** 2020-07-10

**Authors:** Yang Yang, Dan Xing, Yawei Wang, Haobo Jia, Bing Li, Jiao Jiao Li

**Affiliations:** 1 Department of Orthopaedics, Tianjin Hospital, Tianjin, 300211 China; 2 Arthritis Clinic & Research Center, Peking University People’s Hospital, Peking University, Beijing, 100044 China; 3 Department of Electromyography, Tianjin Hospital, Tianjin, 300211 China; 4 Kolling Institute, Faculty of Medicine and Health, University of Sydney, St Leonards, NSW 2065 Australia; 5 School of Biomedical Engineering, Faculty of Engineering and IT, University of Technology Sydney (UTS), Ultimo, NSW 2007 Australia

**Keywords:** Osteoarthritis, Chondrocytes, Long noncoding RNA, HOTAIR, WIF-1, Wnt/β-catenin pathway

## Abstract

**Background:**

Long noncoding RNAs (lncRNAs) are recently found to be critical regulators of the epigenome. However, our knowledge of their role in osteoarthritis (OA) development is limited. This study investigates the mechanism by which HOTAIR, a key lncRNA with elevated expression in OA, affects OA disease progression.

**Results:**

HOTAIR expression was greatly elevated in osteoarthritic compared to normal chondrocytes. Silencing and over-expression of HOTAIR in SW1353 cells respectively reduced and increased the expression of genes associated with cartilage degradation in OA. Investigation of molecular pathways revealed that HOTAIR acted directly on Wnt inhibitory factor 1 (WIF-1) by increasing histone H3K27 trimethylation in the WIF-1 promoter, leading to WIF-1 repression that favours activation of the Wnt/β-catenin pathway.

**Conclusions:**

Activation of Wnt/β-catenin signalling by HOTAIR through WIF-1 repression in osteoarthritic chondrocytes increases catabolic gene expression and promotes cartilage degradation. This is the first study to demonstrate a direct link between HOTAIR, WIF-1 and OA progression, which may be useful for future investigations into disease biomarkers or therapeutic targets.

## Background

Osteoarthritis (OA) is a leading cause of chronic disability worldwide, affecting over 50% of patients above 55–80 years of age [1]. Pain and reduced mobility in OA patients bring much more than a drastic decline in quality of life, but also increased risk of premature death due to cardiovascular disease, diabetes mellitus, obesity, and cognitive disorders [2]. Unfortunately, OA has no cure and current treatments can only relieve symptoms rather than stop or reverse disease progression [3]. A major hurdle preventing the development of effective, disease-modifying treatments for OA is that a full understanding of the pathological mechanisms contributing to OA progression has not been achieved. These likely involve a multitude of complex and interrelated processes affecting the entire joint, including articular cartilage, subchondral bone, synovial tissue and the meniscus [4]. Increasing our understanding of OA pathogenesis may be the key to identifying new disease biomarkers or therapeutic targets to aid the treatment of OA.

The human genome is now known to comprise not only protein-coding elements, which constitute only 2% of the total genetic material present, but also a large amount of genetic material that transcribes multiple families of noncoding RNAs. Many of these noncoding RNAs have been shown to modulate gene expression and have structural, regulatory, or unknown functions [5]. There are two major groups of noncoding RNAs based on their length, short noncoding RNAs and long noncoding RNAs. MicroRNAs are the most commonly studied short noncoding RNAs with a range of roles in affecting cell fate and disease pathophysiology [6]. On the other hand, the role of long noncoding RNAs (lncRNAs) as critical regulators of biological processes, and their effects on tissue development and disease has only begun to emerge within the last decade. LncRNAs are defined as transcripts > 200 nucleotides in length, and are mostly produced by the same transcriptional machinery as messenger RNAs (mRNAs) [7]. LncRNAs are now known to be differentially expressed in many human diseases including metabolic, cardiovascular, neurodegenerative and psychiatric diseases [8], as well as cancer [9]. Although less well studied as in other tissues, lncRNAs have been reported to play critical roles in the development of bone and cartilage, and diseases associated with these tissues [10]. A small number of recent reviews have summarised the relation between lncRNAs and regulation or pathogenesis of OA, including their roles in extracellular matrix degradation, inflammation, chondrocyte and synoviocyte apoptosis, and angiogenesis [11–14]. To date, limited studies have revealed the regulatory roles of specific lncRNAs in OA, including GAS5 [15], lncRNA-CIR [16], and H19 [17] as the top candidates.

Thousands of lncRNAs are shown to be differentially expressed between OA and normal cartilage obtained from patients with knee OA [18]. Our previous study also identified 121 up- or down-regulated lncRNAs in OA compared with normal human cartilage, through microarray analysis that was validated by RT-PCR [19]. From these, HOX antisense intergenic RNA (HOTAIR) was identified as the lncRNA with the most upregulated expression in OA samples (> 20 fold compared to normal samples). General over-expression of HOTAIR is known to induce genome-wide targeting of polycomb repressive complex 2 (PRC2), leading to altered methylation of histone H3 lysine 27 (H3K27) and changes in gene expression [10]. Much evidence suggests that misregulation of HOTAIR is associated with a variety of cancers and cancer metastasis [20–23]. However, the role of HOTAIR in rheumatic diseases is not well understood, with some evidence suggesting that HOTAIR has an important regulatory role in rheumatoid arthritis [24], and that it promotes the expression of ADAMTS-5 and matrix metalloproteinases (MMPs) in osteoarthritic chondrocytes [25, 26]. However, a clear link between the regulatory role of HOTAIR and OA pathogenesis has not been established.

The Wnt/β-catenin signalling pathway is an evolutionarily conserved pathway with critical roles in tissue development and maintenance [27]. In non-regenerating organs such as the mammalian heart, this pathway is quiescent but can be activated in response to injury [28]. A growing body of evidence suggests that this pathway is involved in fibrotic processes as part of the imperfect healing in these organs [27]. Similarly, aberrant activation of Wnt/β-catenin signalling has been linked to the development of OA [29].

Wnt inhibitory factor 1 (WIF-1) is a key inhibitor of the Wnt/β-catenin pathway. WIF-1 binds directly to extracellular Wnt ligands, preventing their interaction with cell surface receptors and hence leading to the degradation of cytosolic β-catenin by the APC/Axin1 destruction complex [30]. Epigenetic silencing of WIF-1 was shown to be an important mechanism causing aberrant activation of the Wnt/β-catenin pathway in several human cancers [31, 32]. Interestingly, altered HOTAIR expression was shown to be involved in repressing the transcription of WIF-1, thereby activating Wnt/β-catenin signalling in oesophageal squamous cell carcinoma [33]. We therefore hypothesised that HOTAIR may promote the progression of OA through a similar axis, by reducing WIF-1 expression and thus activating the Wnt/β-catenin pathway. This is the first study to demonstrate that the lncRNA HOTAIR directly inhibits WIF-1 expression by increasing histone H3K27 methylation in the promoter region, leading to elevated Wnt/β-catenin signalling that promotes cartilage degradation in OA.

## Results

### Gene expression profile of chondrocytes from normal and OA patients

**Fig. 1:**
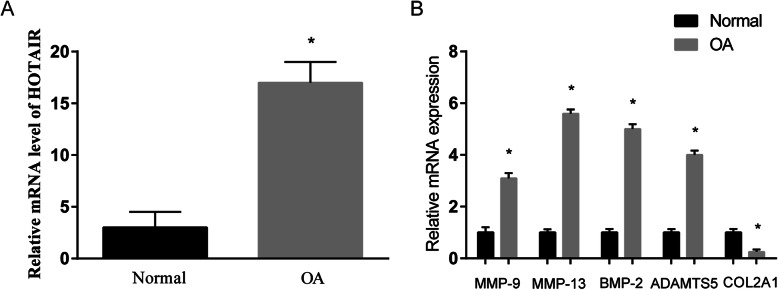
Expression levels of (**a**) HOTAIR and (**b**) OA-related genes in chondrocytes isolated from normal and OA cartilage (*n* = 10). Relative mRNA expression is determined by real-time qRT-PCR and normalised to GAPDH. The OA group is expressed as a fold difference from the normal group. *P < 0.05

OA-related gene expression levels were compared between chondrocytes isolated from knee cartilage of normal and OA patients. OA chondrocytes showed significantly higher HOTAIR expression than normal chondrocytes (Fig. 1a). The osteoarthritic phenotype of OA chondrocytes was confirmed by their significantly elevated expression of matrix degradation markers including MMP-9, MMP-13, BMP-2 and ADAMTS5, as well as reduced expression of COL2A1 which is a major component of the cartilaginous matrix (Fig. 1b).

### Effect of HOTAIR inhibition and over-expression on gene expression in SW1353 cells

**Fig. 2:**
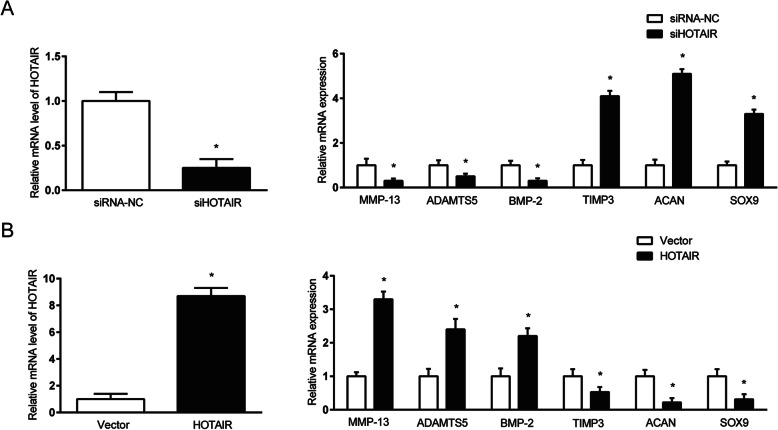
Expression levels of HOTAIR, as well as OA- and cartilage-related genes in SW1353 cells at 48 h after (**a**) siRNA-mediated knockdown of HOTAIR or (**b**) over-expression of HOTAIR by retrovirus infection. Relative mRNA expression is determined by real-time qRT-PCR and normalised to GAPDH. The HOTAIR knockdown and over-expression groups are expressed as a fold difference from their respective controls. *P < 0.05

When HOTAIR function was inhibited in SW1353 cells by siRNA-mediated knockdown of HOTAIR, the mRNA expression of HOTAIR was significantly reduced in these cells (Fig. 2a, left). Accompanying this was a significant reduction in the expression of OA-related catabolic genes MMP-13, ADAMTS5 and BMP-2, as well as significant increase in the expression of cartilage-related anabolic genes TIMP3, ACAN and SOX9 (Fig. 2a, right) compared to the negative control.

Conversely, SW1353 cells induced to over-express HOTAIR by retrovirus infection showed significantly higher mRNA expression of HOTAIR compared to the vector control (Fig. 2b, left). This was accompanied by gene expression changes opposite to those observed for HOTAIR inhibition, that is, significant increase in the expression of MMP-13, ADAMTS5 and BMP-2, as well as significant decrease in the expression of TIMP3, ACAN and SOX9 (Fig. 2b, right).

### Effect of HOTAIR inhibition and over-expression on protein expression in SW1353 cells

**Fig. 3:**
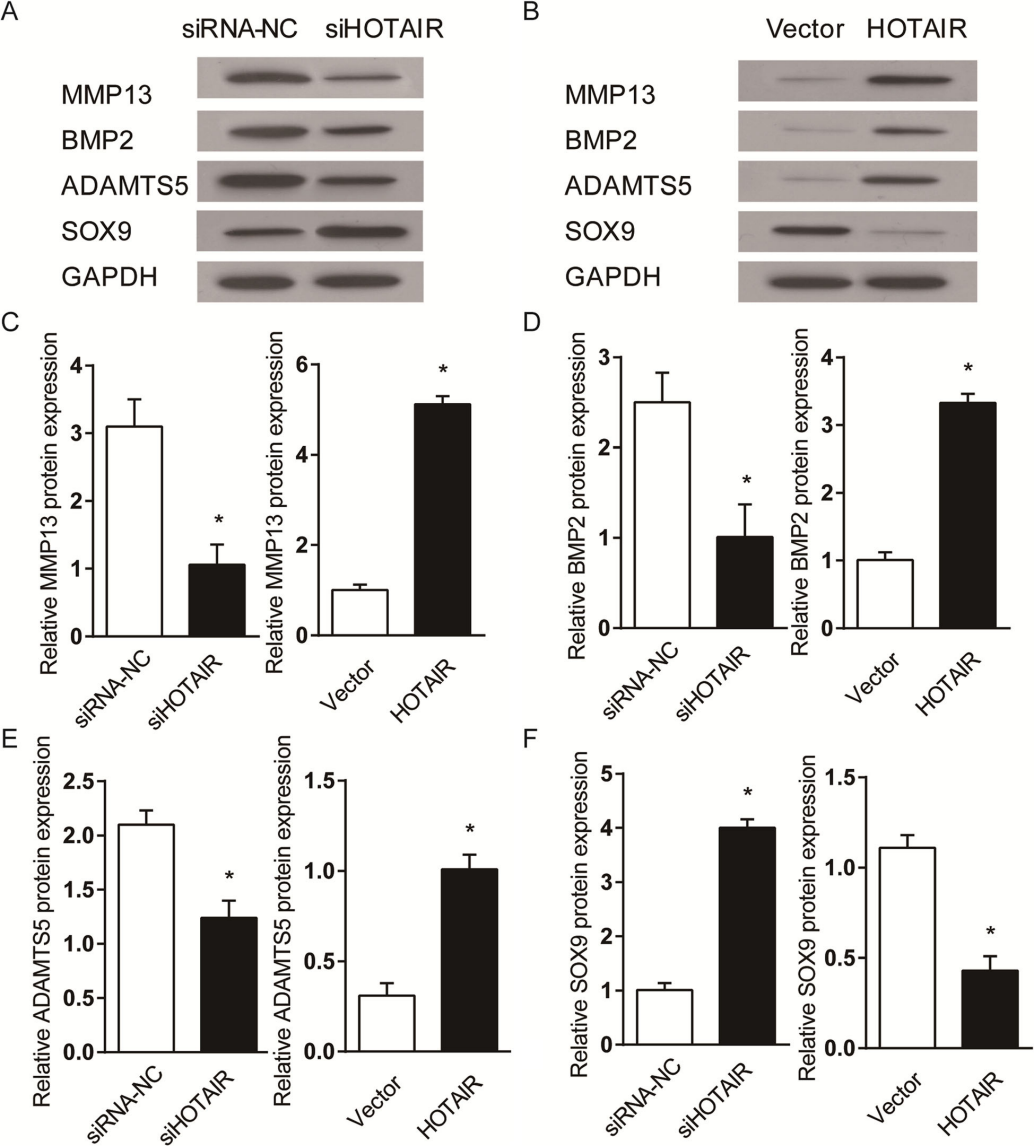
Western blot analysis of OA- and cartilage-related protein expression in SW1353 cells at 48 h after (**a**) siRNA-mediated knockdown of HOTAIR or (**b**) over-expression of HOTAIR by retrovirus infection. Protein expression levels of (**c**) MMP-13, (**d**) BMP-2, (**e**) ADAMTS5 and (**f**) SOX9 are measured by densitometry analysis. The HOTAIR knockdown and over-expression groups are expressed as a fold difference from their respective controls *P < 0.05

Western blot was used to examine the expression of MMP-13, BMP-2, ADAMTS5 and SOX9 at the protein level following HOTAIR knockdown (Fig. 3a) and over-expression (Fig. 3b) in SW1353 cells. The protein expression results aligned with changes in gene expression, where inhibition of HOTAIR function reduced the expression of MMP-13, BMP-2 and ADAMTS5 protein but increased the expression of SOX9 protein (Fig. 3c-f). Meanwhile, over-expression of HOTAIR in SW1353 cells resulted in opposite changes in the expression of these same proteins (Fig. 3c-f).

### HOTAIR regulation of WIF-1 expression

**Fig. 4:**
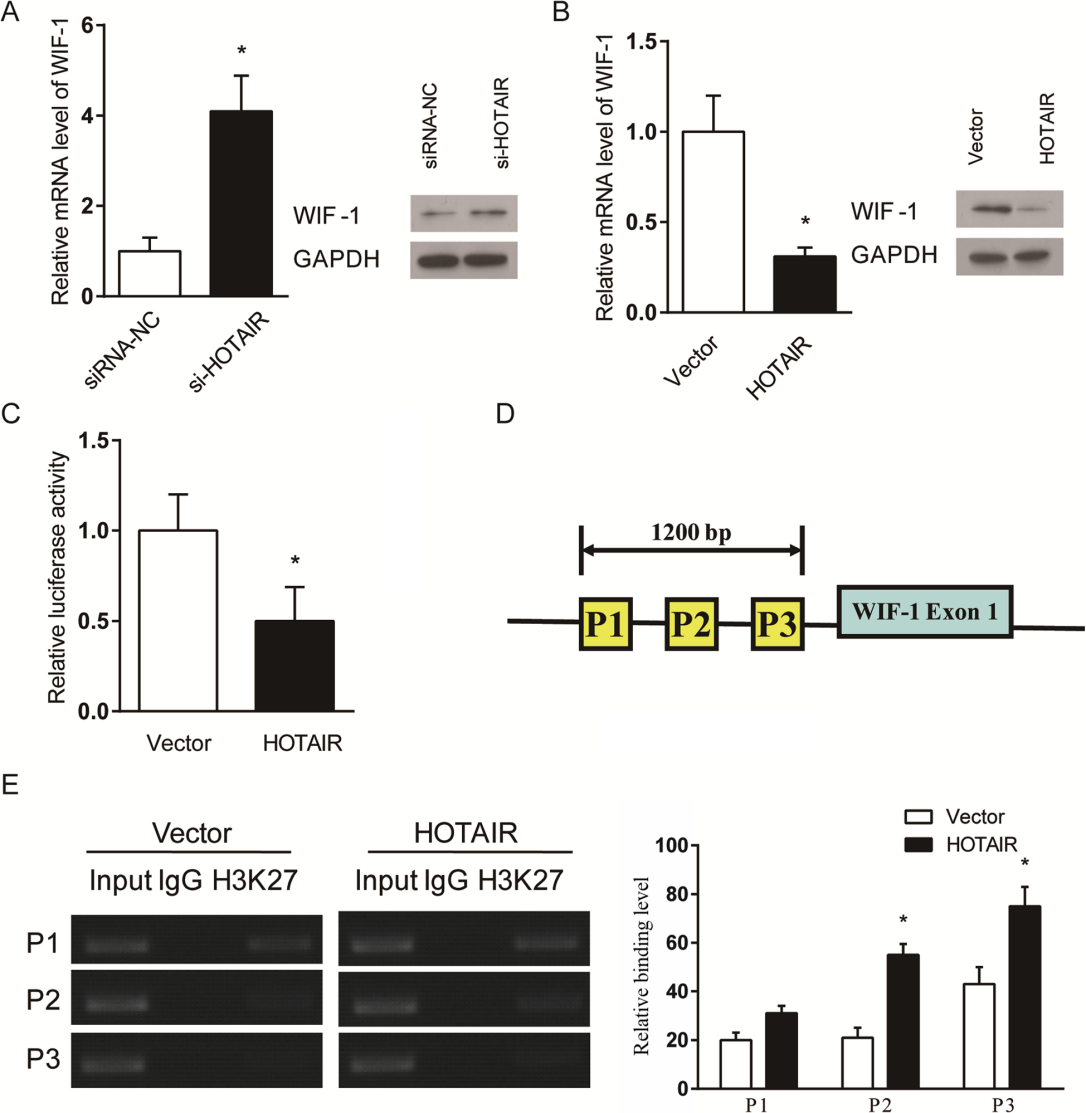
The inverse relation between HOTAIR and WIF-1 expression is demonstrated by WIF-1 mRNA and protein expression in SW1353 cells at 48 h after (**a**) siRNA-mediated knockdown of HOTAIR or (**b**) over-expression of HOTAIR by retrovirus infection, as well as by (**c**) a dual luciferase reporter assay at 36 h after transfecting HOTAIR over-expressing or control SW1353 cells with a vector containing the WIF-1 promoter. The HOTAIR knockdown and over-expression groups are expressed as a fold difference from their respective controls. (**d**) Schematic illustrating primer locations in the WIF-1 promoter. (**e**) ChIP assay showing enrichment of H3K27 at the WIF-1 promoter. The PCR products were analysed by gel electrophoresis. *P < 0.05

To determine the mechanism by which HOTAIR regulates the expression of genes associated with cartilage catabolism and anabolism in OA, we investigated WIF-1 due to its known interactions with HOTAIR in cancer progression. WIF-1 mRNA and protein expression were both greatly elevated in SW1353 cells following siRNA knockdown of HOTAIR (Fig. 4a). Conversely, HOTAIR over-expression in SW1353 cells caused significant reduction in mRNA and protein expression of WIF-1 (Fig. 4b). The inverse relation between HOTAIR and WIF-1 expression was further confirmed through a dual luciferase reporter assay. When transfected with a vector containing the WIF-1 promoter region, SW1353 cells over-expressing HOTAIR showed significant inhibition of luciferase activity compared to control cells (Fig. 4c).

The regulatory mechanism by which HOTAIR modulates WIF-1 expression was investigated by measuring the levels of histone H3K27 trimethylation in SW1353 cells over-expressing HOTAIR. A schematic illustrates targeted primer locations in the WIF-1 gene (Fig. 4d). A chromatin immunoprecipitation (ChIP) assay was used to quantify enrichment of H3K27 in the WIF-1 promoter, which showed a significant increase in H3K27 trimethylation in SW1353 cells over-expressing HOTAIR compared to control cells (Fig. 4e).

### Effects of HOTAIR on Wnt/β-catenin signalling pathway

**Fig. 5:**
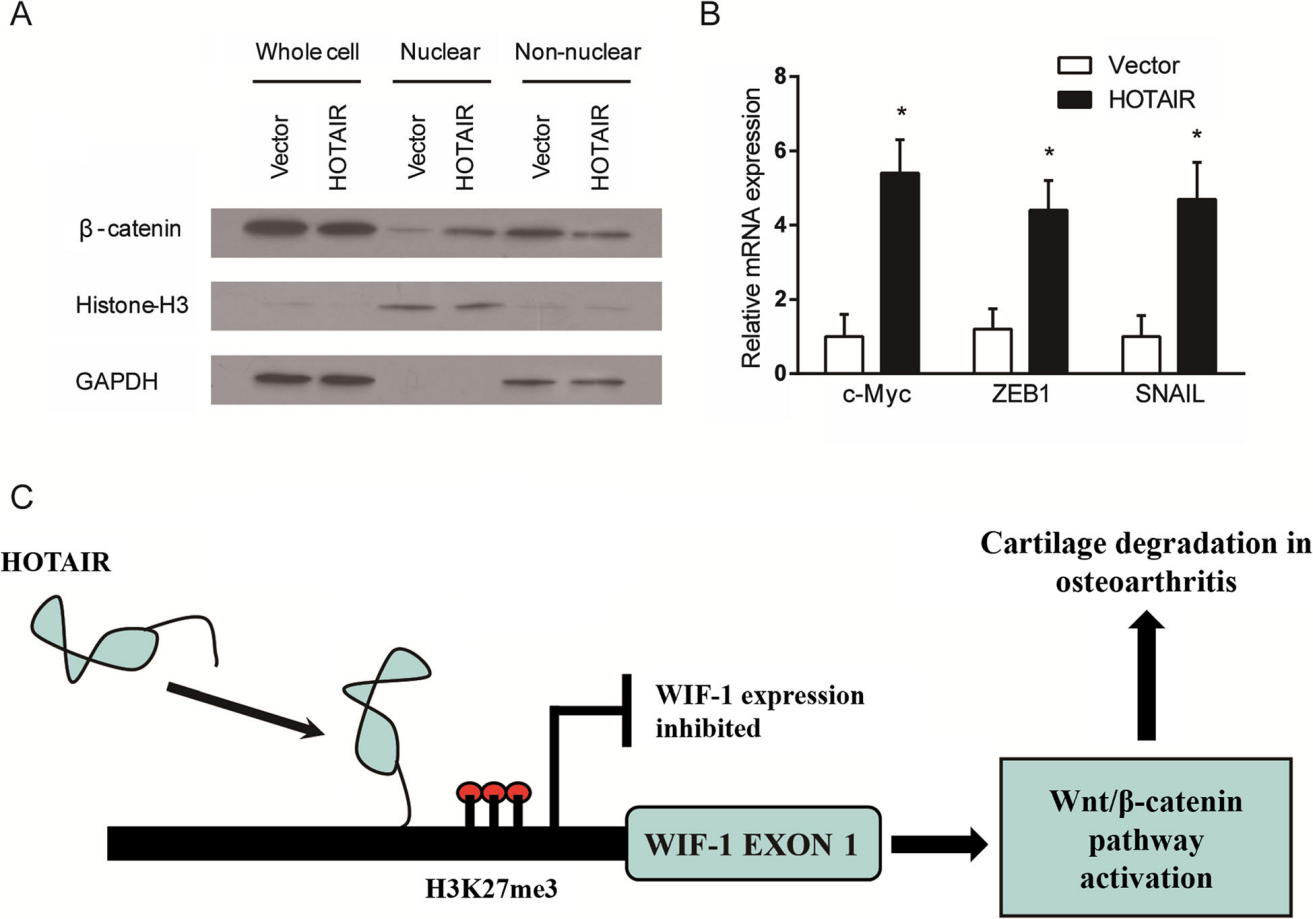
**a** β-catenin protein expression in the whole cell, nucleus and cytoplasm of SW1353 cells at 48 h after over-expression of HOTAIR by retrovirus infection compared to control cells. Histone-H3 was used as a nuclear protein control and GAPDH as a cytoplasmic protein control. **b** Expression levels of c-Myc, ZEB1 and SNAIL as downstream targets of β-catenin signalling in SW1353 cells over-expressing HOTAIR and controls. **c** Schematic of the proposed mechanism by which HOTAIR contributes to cartilage degradation in OA

Since WIF-1 is a key regulator of the Wnt/β-catenin pathway, we proceeded to investigate the effects of HOTAIR expression on Wnt/β-catenin signalling. In SW1353 cells over-expressing HOTAIR, β-catenin concentration was increased in the nucleus and reduced in the cytoplasm compared to control cells (Fig. 5a). Furthermore, the expression levels of c-Myc, ZEB1 and SNAIL as downstream target genes of Wnt/β-catenin signalling were all significantly increased in SW1353 cells over-expressing HOTAIR (Fig. 5b). These results collectively suggested that HOTAIR expression led to accumulation of β-catenin in the cell nucleus and hence activation of the canonical Wnt/β-catenin pathway. The proposed mechanism by which HOTAIR expression affects Wnt/β-catenin signalling in OA development is summarised in a schematic (Fig. 5c).

## Discussion

The pathogenesis of OA involves many complex and interrelated processes that result in local inflammation and degenerative changes in all tissue types within the joint [34, 35]. Structural degradation of the extracellular matrix in key joint tissues, in particular the articular cartilage, is a hallmark of OA progression and is closely related to altered gene expression in chondrocytes and other cells [36]. Over the last decade, studies examining novel pathways in OA pathogenesis have pointed to the important role of epigenetic mechanisms in modulating cell phenotype and gene expression in OA [37–39]. In particular, various microRNAs are now understood to have critical roles in maintaining cartilage homeostasis and have altered expression either as a cause or consequence of OA [38, 40]. However, the roles of lncRNAs in OA pathogenesis are much less well understood. LncRNAs are now being increasingly studied due to growing evidence pointing to their roles in chromatin modification and transcriptional regulation, leading to diverse biological effects including cell cycle regulation, immune surveillance, and cell pluripotency [41]. Although some studies including our own have revealed key lncRNAs associated with OA development [18, 19], much work remains to be done in elucidating the specific regulatory roles of these lncRNAs in OA and the molecular mechanisms involved. This is the first study to show that HOTAIR, one of the most highly upregulated lncRNAs in osteoarthritic chondrocytes [19], acts directly on WIF-1 to modulate the Wnt/β-catenin pathway in OA and provide new insight into its pathophysiological functions.

To understand the link between HOTAIR and OA-related gene expression, we studied the effects of HOTAIR silencing and over-expression in chondrocytes by using SW1353 cells as an established chondrocytic cell model and a reliable transfection host [42]. Although SW1353 is a chondrosarcoma cell line, the cells exhibit similar responses as primary chondrocytes to a variety of catabolic cytokines relevant to OA, and have been used as the cell line of choice in many studies to investigate the molecular mechanisms of OA pathogenesis [43–45]. We found that HOTAIR over-expression in SW1353 cells matched the expression profile of OA-related genes in human primary osteoarthritic chondrocytes, characterised by the upregulation of catabolic enzymes (MMP-13, ADAMTS5) and cartilage repair markers (BMP-2), and downregulation of anabolic markers (TIMP3, SOX9) and cartilage matrix proteins (ACAN). Conversely, silencing of HOTAIR in SW1353 cells resulted in opposite trends in OA-related gene and protein expression. These findings suggested an essential role of HOTAIR in regulating cartilage homeostasis by controlling the expression of key genes and proteins involved in cartilage degradation and restoration.

The roles of WIF-1 in skeletal tissue development have been investigated in other studies. WIF-1 is shown to be a multifunctional modulator of signalling pathways in cartilage development [46, 47], and has differential expression during neonatal and pre-adolescent development in chondrocytes surrounding cartilage canals and the osteochondral junction [48]. Moreover, WIF-1 is shown to have a protective effect against cartilage degradation in experimental arthritis, and has important effects in promoting the balance of cartilage and bone turnover [49]. Although WIF-1 expression levels in articular cartilage are negatively correlated with disease severity in patients with knee OA [50], the mechanisms by which WIF-1 is regulated during OA development have not been previously investigated.

Epigenetic disruptions, some of which lead to gene silencing through methylation of CpG sites in the promoter region and histone modification of genes, have been identified as key events in OA development [37]. However, the upstream and downstream mechanisms associated with these aberrant disruptions remain unclear in the majority of OA-related pathways. In this study, we found that HOTAIR over-expression reduced WIF-1 gene and protein expression by increasing its histone H3K27 methylation in the promoter region, which promoted the activation of the Wnt/β-catenin pathway. This regulatory mechanism was similar to an axis that contributed to metastasis in oesophageal squamous cell carcinoma [33]. Moreover, it was reported that HOTAIR could bind PRC2 to reprogram the chromatin state, thereby regulating the expression of hundreds of genes to promote cancer metastasis [21, 22]. The PRC2 complex is one of the two classes of polycomb-group proteins (PcG) that lead to transcriptional repression by catalysing H3K27 trimethylation [51]. Based on the findings of this study, we propose that HOTAIR may bind with PRC2 to induce histone H3K27 trimethylation in the WIF-1 promoter, which inhibits WIF-1 expression. This leads to increased activation of Wnt/β-catenin signalling that is associated with the progression of OA by inducing excessive cartilage remodelling and degradation [52]. The direct interaction of HOTAIR with the PRC2 complex in this axis needs to be confirmed in human articular chondrocytes, as well as possible interactions relating to H3K27 methylation (such as H3K4 modifications) [53]. The upstream regulatory mechanisms of HOTAIR expression in OA also need to be determined, which may involve interactions with other lncRNAs [54] or mechanoresponsive pathways [55]. Furthermore, histone methylation is known to be affected by inhibitors of DNA methylation, such as 5-Aza-2′-deoxycytidine which is used as an anti-cancer drug [56, 57], and it would be interesting to investigate the effects of applying such compounds on HOTAIR signalling in OA. These additional findings building on the results of this study will confirm our proposed mechanism, and potentially improve the accuracy of predictive models for the pathogenesis of cartilage damage in OA [58]. Further studies demonstrating the interactions of HOTAIR and WIF-1 in normal and OA human cartilage explants, and physiologically relevant in vivo models of OA need to be performed for this pathway to be used as a target for biomarker or therapeutic development.

There were a few limitations in our study. First, although their gene expression results were presented, TIMP3 and ACAN protein expression could not be obtained in the HOTAIR knockdown and over-expression experiments as their levels were very low in both the control and test groups, which prevented the extraction of reliable results to perform data analysis. Second, the enrichment of H3K27me3 in the WIF-1 promoter region of HOTAIR over-expressing cells as demonstrated in the ChIP assay was not able to be confirmed by Western blot due to low protein expression. Last, c-Myc, ZEB1 and SNAIL mRNA were selected for pathway analysis as downstream targets of β-catenin signalling, as these genes were likely to respond with rapid and significant changes in expression, but other potentially suitable targets exist such as cyclin D1. The protein expression of c-Myc, ZEB1 and SNAIL in whole cell lysate was not verified by Western blot as their expression levels were expected to be low or unreliable, and much less sensitive to change compared to mRNA.

## Conclusions

This study has defined a clear link between the expression of the lncRNA HOTAIR and OA development, through its actions on WIF-1 that lead to changes in Wnt/β-catenin signalling. This is the first study to demonstrate that in osteoarthritic cartilage, elevated HOTAIR expression leads to silencing of WIF-1 through H3K27 methylation in the WIF-1 promoter region. Our study provides new evidence for the regulatory role of HOTAIR in OA pathogenesis, which will be useful for future studies investigating the epigenetics of OA and finding effective disease biomarkers or therapeutic targets.

## Methods

### Tissue samples and chondrocyte isolation

This research was approved by the Ethics Committee of Tianjin Hospital, China (2014–008). Discarded cartilage tissues were obtained from 10 normal patients (without OA) undergoing traumatic above-knee amputation and 10 OA patients undergoing total knee replacement surgery, aged 47–78 years. The OA patients were clinically diagnosed to be Kellgren-Lawrence grade 3 based on radiographic examination. All clinical specimens were obtained after patients gave informed consent. The two groups were paired and cartilage samples were matched by age, sex and body mass index.

Chondrocytes were isolated from the articular cartilage of clinical specimens obtained from normal and OA patients. Cartilage samples were minced and digested in 0.15% (w/v) collagenase (CLS-2, Worthington, USA) for 16 h at 37 °C, in medium consisting of Dulbecco’s Modified Eagle Medium (DMEM, Gibco, UK) supplemented with 10% fetal bovine serum (FBS, HyClone, USA), 100 U/mL penicillin (Gibco) and 100 μg/mL streptomycin (Gibco). Isolated chondrocytes were washed in PBS and filtered through a 100 μm cell strainer (BD Biosciences, USA). The cells were seeded at high density (1 × 10^4^ cells/cm^2^) and kept in maintenance medium for 2 days prior to gene expression analysis.

### SW1353 cell culture and transfection

Human chondrosarcoma cells (SW1353) were obtained from the American Type Culture Collection (ATCC). Cells were grown in maintenance medium consisting of DMEM supplemented with 10% FBS at 37 °C with 5% CO_2_.

To silence HOTAIR function, SW1353 cells were transfected with small interfering RNA (siRNA) oligonucleotides targeting HOTAIR or the negative control (50 nM), using Lipofectamine™ RNAiMAX (Invitrogen, USA) according to the manufacturer’s instructions. The gene-specific siRNA is siHOTAIR (5′-GAACGGGAGUACAGAGAGAUU-3′). Transfected cells were kept in maintenance medium for 48 h prior to further analyses.

To overexpress HOTAIR in SW1353 cells, a HOTAIR expression retrovirus vector was constructed. Full-length HOTAIR was amplified by PCR and cloned into the pBABE retroviral vector (Cell Biolabs, USA) using the primers 5′-GACTCGCCTGTGCTCTGGAGCT-3′ and 5′-TTGAAAATGCATCCAGATTTTT-3′. SW1353 cells were infected with retrovirus containing HOTAIR or negative control (vector) in the presence of 10 μg/mL polybrene (Sigma-Aldrich, USA). The supernatant was removed after 24 h and replaced with maintenance medium containing 1 μg/mL puromycin. Cells were cultured for 48 h prior to further analyses.

### Analyses of HOTAIR-overexpressing SW1353 cells

A chromatin immunoprecipitation (ChIP) assay was conducted for SW1353-Vector and SW1353-HOTAIR cells by Tianjin Zhongrui Biotechnology Co. Ltd., using EZ-ChIP™ (catalogue number 17–371; Sigma-Aldrich, USA) according to the manufacturer’s instructions. Briefly, sheared crosslinked chromatin was immunoprecipitated with anti-H3K27me3 antibody (catalogue number 07–449, Sigma-Aldrich) and normal rabbit IgG (catalogue number 12–370, Sigma-Aldrich). After chromatin immunoprecipitation, retrieved DNA was detected by standard end-point PCR. The PCR products were analysed by gel electrophoresis and quantitated using GelAnalyzer. Primers for the WIF-1 promoter region are designed according to published methods [59], and the sequences are listed in Supplementary Table 1.

The nuclear and cytoplasmic protein fractions were extracted from SW1353-Vector and SW1353-HOTAIR cells using the NE-PER™ Nuclear and Cytoplasmic Extraction Kit (catalogue number 78833; Thermo Fisher Scientific, USA) according to the manufacturer’s instructions. Proteins were detected by Western blot analysis.

### Dual luciferase reporter assay

pGL3-WIF1 Promoter was constructed by inserting PCR product containing 1205 bp in the 5′-flanking sequence of the human WIF-1 promoter into the pGL3-basic vector. Together with 40 ng pRL-TK Vector (Promega, USA) containing Renilla luciferase as an internal control, 200 ng pGL3-WIF1 Promoter were transfected into SW1353-Vector and SW1353-HOTAIR cells using Lipofectamine™ 2000 (Invitrogen, USA) according to the manufacturer’s instructions. Transfected cells were cultured in maintenance medium, and luciferase reporter assays were performed 36 h after transfection. Cells were lysed and the firefly and Renilla luciferase activities in each well were measured using the Dual Luciferase Reporter Assay System (Promega). All measured luciferase activities were normalised to pRL-TK Vector activity, and firefly luciferase activity was normalised to Renilla luciferase activity for each well.

### Gene expression analysis

Total RNA was isolated from cells using the TRIzol reagent (Invitrogen) according to the manufacturer’s instructions. Briefly, samples were homogenised using the TRIzol reagent and chloroform was added, after which RNA was precipitated using isopropanol. The RNA was resuspended in 20 μL purified water.

Reverse transcription into cDNA was performed using 1 μg total RNA from each sample using PrimeScript RT reagent Kit with gDNA Eraser (Takara Bio, USA) according to the manufacturer’s instructions. Gene expression levels were quantified with SYBR Premix Ex TaqII kit (Takara Bio) using a 7900HT Fast Real-Time PCR System (Applied Biosystems, USA) and normalised to GAPDH. Primer sequences were purchased from Sigma-Aldrich and listed in Supplementary Table 1. Relative gene expression was calculated using the comparative Ct (2^−ΔΔCT^) method.

### Western blotting

Cells were lysed using a cell lysis buffer (Beyotime, China), and protein content was quantified using a BCA assay (Thermo Fisher Scientific). Equal amount of protein from each sample was electrophoresed on 10% (w/v) SDS-PAGE and transferred to PVDF membranes (EMD Millipore). The membranes were blocked with 5% milk in TBST for 1 h at room temperature, and incubated at 4 °C overnight with primary antibodies against WIF-1 (catalogue number sc-373780; Santa Cruz Biotechnology, USA), β-catenin (catalogue number C7082; Sigma-Aldrich), and GAPDH (catalogue number 5174; CST, China) at 1/1000 dilution. Membranes were washed three times for 10 min each with TBST, and then incubated for 1 h at room temperature with secondary antibody conjugated to horseradish peroxidase (HRP) at 1/5000 dilution (anti-rabbit IgG, CST). The blots were visualised using enhanced chemiluminescence reagent (ECL, Thermo Fisher Scientific, USA). The blot intensity was quantified using molecular imaging software (Carestream Health, USA) after normalising to the corresponding loading control.

### Statistical analysis

Data for all experiments were obtained from at least three independent samples, and all results were expressed as mean ± standard deviation. Statistical analysis was performed using the Stata 12.0 statistical software package (StataCorp, USA). One-way ANOVA with Tukey’s multiple comparisons test was used for statistical comparisons. A *P*-value of 0.05 was considered statistically significant.

## Supplementary information

**Additional file 1: Supplementary Table 1.** Primer sequences

**Additional file 2.**

## Data Availability

The datasets used and/or analysed during the current study are available from the corresponding author on reasonable request.

## Abbreviations

ATCC: American Type Culture Collection
ChIP: Chromatin immunoprecipitation
DMEM: Dulbecco’s Modified Eagle Medium
FBS: Fetal bovine serum
H3K27: H3 lysine 27
HRP: Horseradish peroxidase
HOTAIR: HOX antisense intergenic RNA
lncRNA: Long noncoding RNA
MMP: Matrix metalloproteinase
mRNA: Messenger RNA
OA: Osteoarthritis
PRC2: Polycomb repressive complex 2
WIF-1: Wnt inhibitory factor 1
