# Supplementary material for: A long non-coding RNA, HOTAIR, promotes cartilage degradation in osteoarthritis by inhibiting WIF-1 expression and activating Wnt pathway
Source: BMC Mol Cell Biol. 2020 Jul 10;21:53. doi: 10.1186/s12860-020-00299-6 (PMC7350747; doi:10.1186/s12860-020-00299-6)
Supplement: Supplementary file 1 — Additional file 1: Supplementary Table 1. Primer sequences [file 12860_2020_299_MOESM1_ESM.docx]

**Supplementary Table 1.** Primer sequences

| **Primers for RT-PCR** | |
| --- | --- |
| GAPDH forward primer | TGCACCACCAACTGCTTAGC |
| GAPDH reverse primer | GGCATGGACTGTGGTCATGAG |
| MMP-9 forward primer | TGTACCGCTATGGTTACACTCG |
| MMP-9 reverse primer | GGCAGGGACAGTTGCTTCT |
| MMP-13 forward primer | ACTGAGAGGCTCCGAGAAATG |
| MMP-13 reverse primer | GAACCCCGCATCTTGGCTT |
| BMP-2 forward primer | ACCCGCTGTCTTCTAGCGT |
| BMP-2 reverse primer | TTTCAGGCCGAACATGCTGAG |
| ADAMTS5 forward primer | GAACATCGACCAACTCTACTCCG |
| ADAMTS5 reverse primer | CAATGCCCACCGAACCATCT |
| COL2A1 forward primer | TGGACGCCATGAAGGTTTTCT |
| COL2A1 reverse primer | TGGGAGCCAGATTGTCATCTC |
| HOTAIR forward primer | CCATCTTTATGATGAGGCTTGT |
| HOTAIR reverse primer | GCTGAGATAGAGGTGCTTGG |
| TIMP3 forward primer | CATGTGCAGTACATCCATACGG |
| TIMP3 reverse primer | CATCATAGACGCGACCTGTCA |
| ACAN forward primer | ACTCTGGGTTTTCGTGACTCT |
| ACAN reverse primer | ACACTCAGCGAGTTGTCATGG |
| SOX9 forward primer | AGCGAACGCACATCAAGAC |
| SOX9 reverse primer | CTGTAGGCGATCTGTTGGGG |
| WIF-1 forward primer | AGTGTCCTGATGGGTTCCAC |
| WIF-1 reverse primer | TGGTTGAGCAGTTTGCTTTG |
| c-Myc forward primer | GCTGGACCAGATGTATGTCCC |
| c-Myc reverse primer | ATCATTTCCATGACGGCCTGT |
| ZEB1 forward primer | GATGATGAATGCGAGTCAGATGC |
| ZEB1 reverse primer | ACAGCAGTGTCTTGTTGTTGT |
| SNAIL forward primer | TCGGAAGCCTAACTACAGCGA |
| SNAIL reverse primer | AGATGAGCATTGGCAGCGAG |
| **Primers in WIF-1 promoter region** | |
| WIF-1 P1 forward primer | AACCCTTTTGCTTCCGTTTT |
| WIF-1 P1 reverse primer | AAAGTTGCCGAATTCACAGG |
| WIF-1 P2 forward primer | GGCAATTTGCGCCTTCAG |
| WIF-1 P2 reverse primer | CTTCGGGGGAGGGAAATG |
| WIF-1 P3 forward primer | ATGTCCCAGGGGTCTCTGA |
| WIF-1 P3 reverse primer | GAGAACAGAAGAGCGGGAAG |
