# Supplementary figures and images for: A long non-coding RNA, HOTAIR, promotes cartilage degradation in osteoarthritis by inhibiting WIF-1 expression and activating Wnt pathway
Source: BMC Mol Cell Biol. 2020 Jul 10;21:53. doi: 10.1186/s12860-020-00299-6 (PMC7350747; doi:10.1186/s12860-020-00299-6)

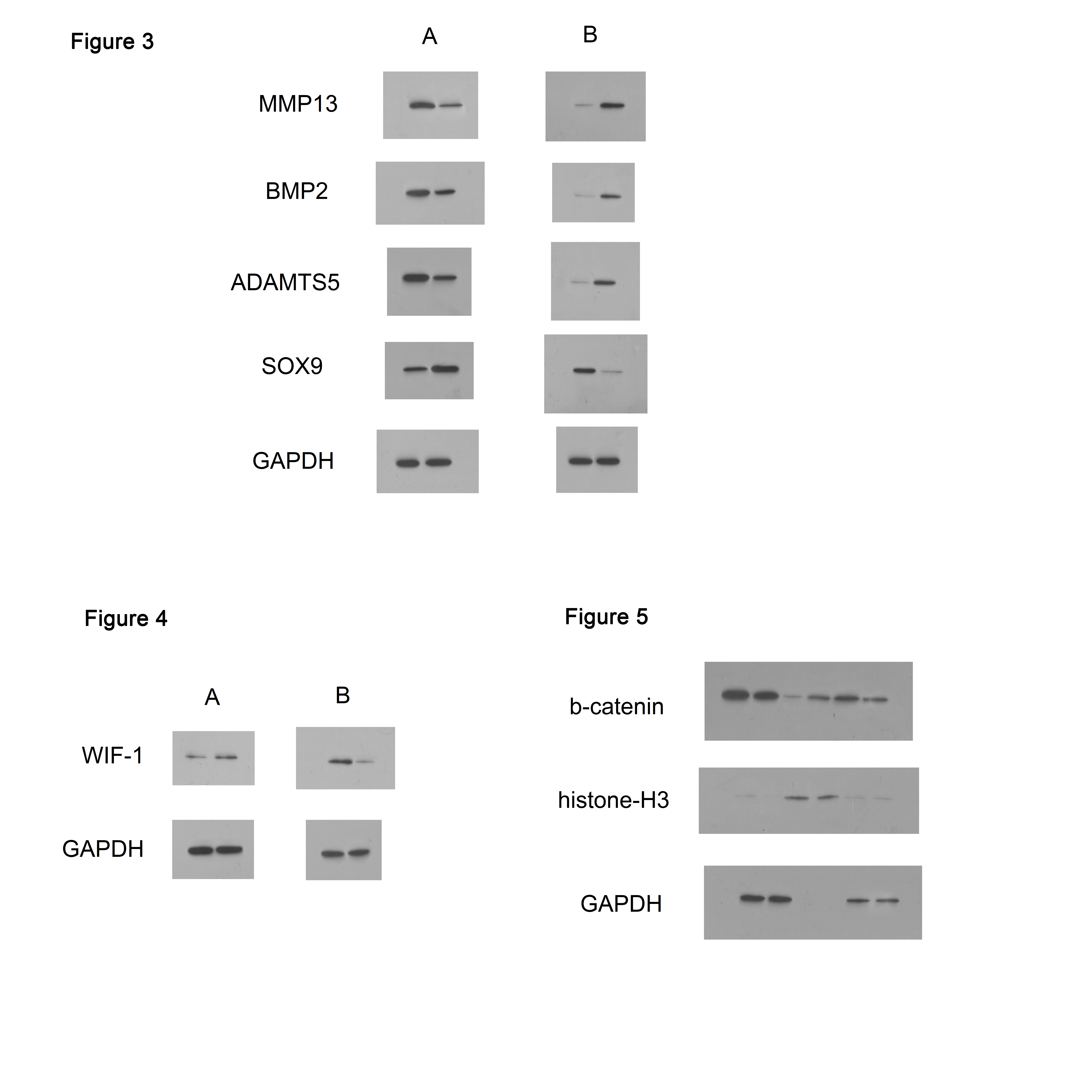

Supplement: Supplementary file 2 — Additional file 2. [file 12860_2020_299_MOESM2_ESM.tif]
